# Paraspeckle nuclear bodies—useful uselessness?

**DOI:** 10.1007/s00018-012-0973-x

**Published:** 2012-04-04

**Authors:** Shinichi Nakagawa, Tetsuro Hirose

**Affiliations:** 1 RNA Biology Laboratory, RIKEN Advanced Science Institute, 2-1 Hirosawa, Wako, Saitama, 351-0198 Japan; 2 Functional RNomics Team, Biomedicinal Information Research Center, National Institute of Advanced Industrial Science and Technology (AIST), 2-4-7 Aomi, Koutou, Tokyo 135-0064 Japan

**Keywords:** Paraspeckles, Nuclear structures, NEAT1, Noncoding RNA

## Abstract

The nucleus of higher eukaryotes, such as humans and mice, is compartmentalized into multiple nuclear bodies, an organization that allows for the regulation of complex gene expression pathways that are characteristic of these organisms. Paraspeckles are recently discovered, mammalian-specific nuclear bodies built on a long, non-protein-coding RNA, NEAT1 (nuclear-enriched abundant transcript 1), which assembles various protein components including RNA-binding proteins of the DBHS (*Drosophila* behavior and human splicing) family. Paraspeckles have been proposed to control several biological processes, such as stress responses and cellular differentiation, but their function at the whole animal level remains unclear. In this review, we summarize a series of studies on paraspeckles that have been carried out in the decade since their discovery and discuss their physiological function and molecular mechanism.

## Introduction

According to a maxim of Zhuangzi, everyone knows the usefulness of what is useful, but few know the usefulness of what is useless. This aphorism may apply to the emerging research field of nuclear bodies termed paraspeckles, in which we have a detailed understanding of the molecular components and their assembly cascade but little knowledge of their physiological relevance in living animals.

The nucleus of higher eukaryotes is not uniform in structure but is functionally divided into multiple compartments or nuclear bodies that contain particular sets of proteins and nucleic acids involved in distinct molecular processes [1–5]. One of the best characterized of the classical nuclear bodies is the nucleolus, where ribosome biogenesis occurs, and this body can be easily recognized under conventional light microscopy. Other nuclear bodies include nuclear speckles, PML bodies, Polycomb bodies, Cajal bodies, histone locus bodies, and nuclear stress bodies [6–9]. These nuclear bodies are usually identified by immuno-histochemical localization of their molecular constituents, which are closely associated with their function. Nuclear speckles contain essential splicing factors, including UsnRNPs and SR-family proteins, and various splicing modulators, suggesting that they are involved in the processing of pre-mRNAs [6]. PML bodies are proteinaceous structures containing PML and various kinds of Sumoylated proteins and are proposed to control a variety of cellular processes including senescence and responses to apoptotic signals and DNA damage [7]. Polycomb bodies comprise the Polycomb-associated chromatin modifier complex and control the epigenetic regulation of gene expression through histone modifications [8]. Cajal bodies (also termed coiled bodies) contain a set of proteins required for the maturation of UsnRNPs and snoRNPs [9]. Histone locus bodies, which often overlap with the Cajal bodies, are involved in the biogenesis of histone mRNAs [9]. The organized configuration of these nuclear bodies is considered essential for the complex regulation of gene expression and the subsequent higher-order biological processes typically found in higher eukaryotes.

Paraspeckles are among the most recently identified nuclear bodies and were first described in 2002 [10, 11]. In this review, we will chronologically review seminal studies on paraspeckles that have been conducted in the past decade and discuss their physiological function.

## Discovery and initial characterization of paraspeckles

During the proteomic identification of nucleolar proteins and the investigation of their subnuclear localization, A. Fox and colleagues [10] in A. Lamond’s laboratory at the University of Dundee, UK, serendipitously found that multiple RNA-binding proteins are co-localized into distinct foci within the nucleus and named these foci paraspeckles because of their close positional association with nuclear speckles. Paraspeckles are found in almost all of the cultured cell lines and primary cultures from tissues [11], except for embryonic stem cells [12]. Initially, three proteins were identified as paraspeckle components, including PSP1 (paraspeckle protein 1), PSP2 (paraspeckle protein 2; also known as COAA, RBM14, SIP, and SYTIP1), and p54^nrb^ (nuclear RNA-binding protein 54 kDa, also known as NONO and NMT55) [10]. Subsequently, PSF (polypyrimidine tract-binding protein-associated splicing factor; also known as SFPQ), a nuclear protein highly homologous to p54^nrb^, was demonstrated to localize to paraspeckles [13, 14]. CFIm68 (cleavage factor Im 68 kDa, also known as CPSF6) [15], and Fus (fused in sarcoma) [16] are coincidentally identified as components of the nuclear bodies. Two transcription factors, Sox9 (SRY-box containing gene 9) [17] and Bcl11a (B-cell chronic lymphoid leukemia 11A) [18], have also been reported to co-localize with paraspeckle markers when overexpressed, although the endogenous proteins do not accumulate at paraspeckles, instead showing punctate but broad distribution throughout the nucleoplasm. The paraspeckle proteins identified thus far are not involved in common cellular processes, and the only functional feature shared by authentic paraspeckle proteins is their RNA-binding activity. In other words, paraspeckles seem to attract a broad range of functionally non-related proteins involved in diverse nuclear processes and separate them from the other regions of the nucleus. This property of paraspeckles might be metaphorical if we consider their physiological roles, as we discuss later. Currently, more than 30 nuclear proteins are known to localize to paraspeckles (T.H., unpublished observations), and further studies may identify further protein components.

Three of the paraspeckle proteins, PSP1, p54^nrb^ and PSF, share a similar domain organization, being composed of two classical RNA recognition motifs (RRMs) followed by a conserved domain termed DBHS (*Drosophila* behavior and human splicing) domain that contains proline-rich coiled-coil motifs [11]. The name DBHS itself does not necessarily represent a characteristic physiological function of the family proteins but is rather derived from the following two independent studies: first, nonA (no-on-transient A), a* Drosophila* homolog of p54nrb, is required for normal vision and courtship songs [19], and second, PSF, which was originally identified as a factor that associates with splicing factor PTB (polypyrimidine-tract binding protein), is considered to also be a splicing factor [20]. Importantly, two DBHS proteins, PSF and p54^nrb^, are essential for the formation and maintenance of paraspeckles, and their depletion leads to disorganization of the paraspeckles [21]. Accordingly, the DBHS family proteins are also termed “core paraspeckle proteins” [11]. PSP1, however, is thought to be dispensable for paraspeckle formation, at least in HeLa cells [21]. DBHS family proteins form hetero-dimers [22], and p54^nrb^ and PSF are often co-purified in biochemical studies to identify factors that bind to specific nucleic acids or protein factors [23–35]. The coiled-coil domain of PSP1 is required for its binding to p54^nrb^, and this interaction is essential for the localization to paraspeckles [14]. The formation of DBHS dimers is specific; PSP1 dimerizes with p54^nrb^ but not with PSF, and PSF also forms a heterodimer with p54^nrb^ [14]. Recently, a PSP1-p54^nrb^ heterodimer has been crystallized [36], and further analysis will reveal the structural-functional relationships of DBHS family proteins.

## Identification of the architectural RNA component of paraspeckles—lncRNAs NEAT1 is essential for paraspeckle formation

**Fig. 1:**
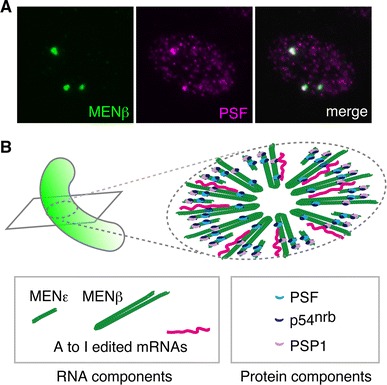
The nuclear body paraspeckle and its provisional ultrastructure model. **a** Expression of NEAT1 (*green*) and PSF (*magenta*) in MEFs. Each nucleus typically contains 5–10 paraspeckles. **b** Provisional ultrastructure model of paraspeckles. NEAT1 transcripts are radially arranged perpendicular to the longer axis of paraspeckles with the 5′ and 3′ ends of the transcripts facing outward. Note that the precise stoichiometry of each paraspeckle components is not known

Since the early stages of paraspeckle studies, the nuclear bodies have been recognized to be sensitive to transcriptional inhibition and RNase treatment, suggesting that certain ribonucleic acids play a structural role [10, 14]. In 2005, K. Prasanth and colleagues in D. Spector’s laboratory at the Cold Spring Harbor Laboratory, USA, identified A-to-I edited CTN RNA, a long isoform transcribed from mCAT2 (mouse cationic amino acid transporter 2), as the first RNA component of paraspeckles. Although they proposed that hyper A-to-I edited RNAs are a major functional target of the nuclear bodies (see the discussion in the following section) [13], the removal of CTN-RNA by antisense oligonucleotides did not lead to disruption of the paraspeckles [13], and thus, the architectural RNA components remained unknown. In 2007, A. Chess and colleagues at Harvard Medical School, USA, re-characterized NEAT1 (nuclear-enriched abundant transcript 1) and NEAT2, which had previously been identified as VINC (virus inducible noncoding RNA) [37] and Malat1 (metastasis associated lung adenocarcinoma transcript 1) [38], respectively, as abundant nuclear long non-protein-coding RNAs (lncRNA) [39]. They demonstrated that Malat1/NEAT2 localizes to nuclear speckles and reported that NEAT1 localizes to distinct nuclear bodies that closely associate with nuclear speckles; i.e., presumptive paraspeckles, although this was not clearly mentioned in the paper [39] (Fig. 1). In 2009, four independent research groups headed by T. Hirose (National Institute of Advanced Industrial Science and Technology, Japan), D. Spector, J. Lawrence (University of Massachusetts Medical Center, USA), and G. Carmichael (University of Connecticut, USA) reported almost simultaneously that NEAT1 plays architectural roles during the formation of paraspeckles [12, 21, 40, 41]. These studies demonstrated that paraspeckles are disintegrated upon depletion of NEAT1 transcripts by an antisense oligonucleotide or siRNA, and their protein components become evenly distributed throughout the nucleoplasm.

The NEAT1 locus generates short (3.7 kb in human; 3.2 kb in mouse) and long (23 kb in human; 20 kb in mouse) noncoding RNA isoforms [21], which had been described as MENε and MENβ, two of the 33 transcripts identified in a large genomic region associated with Type I multiple endocrine neoplasia (MEN) [42]. MENε and MENβ are not involved in the pathology of MEN, and the current HUGO official nomenclature for MENε and MENβ is NEAT1_1 and NEAT1_2, respectively. Despite the observation that both of the NEAT1 transcripts are synthesized by RNA polymerase II, each isoform possesses a distinct structure at its 3′ terminus; NEAT1_1 is canonically poly-adenylated, whereas NEAT1_2 lacks usual poly-A tail, with its 3′ terminus being processed by RNaseP cleavage [40]. Importantly, NEAT1_2, but not NEAT1_1, play an architectural role in the formation of paraspeckles [21, 40], although both transcripts localize to the paraspeckles and associate with DBHS family proteins. This conclusion is supported by the following observations: first, the specific depletion of NEAT1_2 leads to the disappearance of or a reduction in the number of paraspeckles [21, 40]; second, the disruption of the paraspeckles upon knockdown of PSF or p54^nrb^ is accompanied by a dramatic downregulation of NEAT1_2, whereas the level of NEAT1_1 is affected only modestly [21]; and third, the majority of the cells in mouse tissues express NEAT1_1 but not NEAT1_2, and paraspeckle markers do not make prominent foci in these cells [43]. In addition, NEAT1_2, but not NEAT1_1, can rescue the formation of paraspeckles in primary mouse embryonic fibroblast cells (MEFs) obtained from NEAT1 knockout mice (T.H. and S.N., unpublished observation), further supporting the indispensable role of NEAT1_2 for the formation of paraspeckles. In contrast, overexpression of NEAT1_1 resulted in an increased number of paraspeckles [41], suggesting that NEAT1_1 does have paraspeckle-forming activity under certain conditions or that NEAT1_1 increases the efficiency of paraspeckle formation in cooperation with its longer isoform, NEAT1_2.

## Assembly of paraspeckle protein components on NEAT1 and their organization

**Fig. 2:**
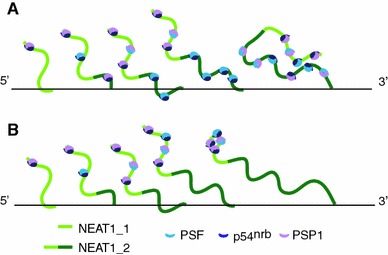
Co-transcriptional assembly of paraspeckle components at the NEAT1 transcription site. Paraspeckle components bind to newly synthesized NEAT1 transcripts at the transcription site to form paraspeckles. The transcript of NEAT1_2, the longer isoform of NEAT1, may function by either providing more entry sites for the paraspeckle components (**a**) or tethering NEAT1_1 at the transcription site for a longer period, allowing sufficient time for the nuclear bodies to form (**b**)

Following the discovery of the architectural RNA component NEAT1 and the core structural protein components of paraspeckles, D. Spector and colleagues [44] reported direct visualization of the paraspeckle component assembly process. One of the key issues regarding the mechanism of nuclear body formation is whether the molecular components assemble in a random, self-organizing manner like the growth of crystals or accumulate around seeding molecules in a distinct, orderly manner like the assembly line of automobiles [45]. Spector and colleagues [44] demonstrated that the immobilization and clustering of any single protein component of paraspeckles results in the failure to build paraspeckles, although partial protein components are specifically recruited to the accumulation site. For example, PSP1 recruits p54^nrb^, probably through its hetero-dimerizing activity but does not assemble any other paraspeckle components [44]. In contrast, the de novo formation of “functional” paraspeckles (i.e., nuclear bodies that can recruit A-to-I edited RNAs) is induced upon conditional expression of NEAT1, and the newly formed paraspeckles are generated around the exogenous transcriptional site as early as 5 min after the induction of ectopic expression [44]. These results clearly indicate that NEAT1 transcripts serve as seeding molecules, leading to the assembly of other components (Fig. 2). Fluorescence recovery after photobleaching (FRAP) analysis revealed that the protein components of the paraspeckles are fairly dynamic and shuttle between the nuclear bodies and the nucleoplasm, whereas NEAT1 transcripts exhibit much slower kinetics [44], further supporting the core architectural function of this noncoding RNA. Interestingly, paraspeckle formation and maintenance are highly dependent on ongoing transcription, and downregulation of exogenous NEAT1 transcription quickly leads to dispersion of de novo formed paraspeckles [44]. This observation is consistent with the previously reported behavior of paraspeckle components upon transcriptional inhibition, which induces the destabilization of NEAT1 transcripts and the redistribution of paraspeckle protein components into a distinct nuclear structure termed the perinucleolar cap [10].

A similar study was reported by M. Dundr and colleagues [46] at the Rosalind Franklin University of Medicine and Science, USA, using a slightly different experimental system. Instead of conditionally inducing the expression of NEAT1, these authors immobilized transcribed NEAT1 onto a distinct chromosome locus using the MS2 RNA-tag system and found that paraspeckle components are recruited to the immobilized site [46]. Therefore, the transcription of NEAT1 and paraspeckle formation are functionally separable, and the assembly of paraspeckle components will start when the concentration of NEAT1 reaches a critical concentration, which normally occurs only at the transcription site. Interestingly, these authors could successfully induce the accumulation of paraspeckle components by immobilizing NEAT1_1, which normally cannot form prominent paraspeckles in the absence of NEAT1_2 [44]. The artificial clustering may allow the accumulation of NEAT1_1 transcripts above the critical threshold concentration, leading to de novo formation of paraspeckle-like granules without ongoing transcription. This mechanism may explain why the transient overexpression of NEAT1_1 resulted in the increased number of paraspeckles in a previous study [41], considering the extraordinarily abundant expression obtained by transient gene transfer methods.

Electron microscopic (EM) analyses further extended our knowledge regarding the structural organization of paraspeckles. Earlier EM studies identified a distinct electron-dense nuclear structure termed IGAZ (interchromatin granule associated zone) that closely associates with interchromatin granules, structures that correspond to nuclear speckles at the light microscopic level [47]. Subsequently, PSF and CFIm68 were demonstrated to localize to the IGAZ [48], suggesting that this structure is essentially equivalent to paraspeckles. Recently, G. Pierron and colleagues [22] at the Institut André Lwoff, France, reported an intriguing spatial organization of NEAT1 transcripts within the paraspeckles at the ultrastructural level using EM in situ hybridization. When probes that detect the 5′- or 3′-end region of NEAT1_2 are used as probes for in situ hybridization, the signals were distributed to the peripheral part of IGAZ [22]. When probes that detect the central region of NEAT1_2 are used, the signals were mainly restricted to the central region of IGAZ. In contrast, protein components such as PSP1 and p54^nrb^ are distributed rather uniformly throughout the IGAZ [22]. The electron-dense feature of IGAZ is largely due to its protein components because paraspeckles become rather electron-lucent after protease treatment [22]. IGAZ was originally identified as an electron-dense structure containing U1 but not U2 snRNA, but U1 snRNA is not particularly concentrated to paraspeckles, at least at the light microscopic level [11]. Interestingly, the length of the shorter axis of IGAZ emerging on the EM cross sections is constrained to less than 360 nm, whereas the size of its longer axis is quite variable [22]. These characteristics of size constraint can be obtained when cylindrical objects of fixed diameter are sectioned. Taken together, paraspeckle components are apparently arranged into a “sausage-like” structure of constant diameter, and NEAT1_2 transcripts are folded in half and radially aligned along the vertical plane with the central region facing toward the center (Fig. 1). Alternatively, NEAT1_2 transcripts might be arranged in a crosswise manner with both ends at the opposite sides of the periphery. Considering that the length of NEAT1_2 is approximately 7 μm (3.4 Å × ~20 kb), the transcripts are highly packed into a compact structure with a packing ratio of 20–40, which is comparable with DNA in the 30-nm fiber in packed nucleosomes. Whichever model is correct, paraspeckles appear to possess highly ordered structures with a regular arrangement of architectural noncoding RNAs and associated protein components.

Although core paraspeckle protein components are considered to bind directly to NEAT1, their precise binding sites have not been fully characterized. Using in vitro mobility shift assays, P. Rangarajan and colleagues [49] at the Indian Institute of Science, India, identified three binding sites for p54^nrb^ in NEAT1_1/VINC, which are located in the 5′ and 3′ regions of the transcript. No conserved sequences or structural motifs are found in the three binding sites, suggesting that p54^nrb^ recognizes a yet unidentified higher order structure of NEAT1_1. Considering that NEAT1_2 is essential for the formation of paraspeckles, a NEAT1_2 specific region may also provide binding sites for core paraspeckle components (Fig. 2a). Alternatively, NEAT1_2 may simply function to “tether” NEAT1_1 for longer periods at the transcription site, providing sufficient time for the assembly of paraspeckle components (Fig. 2b). If the latter is true, then extended transcription at the NEAT1 locus, but not the NEAT1_2 sequence itself, is essential for the assembly of paraspeckle components; this might explain why the artificial tethering of NEAT1_1 at a specific genomic locus leads to paraspeckle formation [46]. It is an intriguing possibility that the non-canonical 3′ terminal structure of NEAT1_2 may contribute the specific function of this isoform. Identifying the functional domain of the NEAT1_2 specific region is essential to discriminate between the two possibilities.

## Proposed function of paraspeckles—nuclear retention of hyper A-to-I edited RNAs

As mentioned above, the first insight into the cellular function of paraspeckles was developed by Prasanth and colleagues [13] in Spector’s laboratory in 2005, through the identification of the paraspeckle-localizing hyper A-to I edited mRNA, CTN-RNA. CTN-RNA is an alternative splicing isoform of mCAT2 and contains extended stretches of 3′ UTRs containing inverted insertions of the retrotransposon SINE (short interspersed nuclear element) [13]. The inverted repeat sequences form intra-molecular double-stranded RNA structures, which are recognized by double-stranded RNA-specific adenosine deaminase (ADAR), resulting in the conversion of adenine to inosine [50]. The hyper-edited CTN-RNAs escape nuclear export and are efficiently retained in the nucleus, where the majority localize to the paraspeckles [13]. The subnuclear localization of CTN-RNA coincides well with the preceding finding that p54^nrb^, one of the core paraspeckle protein components, preferentially recognizes hyper A-to-I edited RNAs over non-modified RNAs [24]. Importantly, cellular stresses such as transcriptional inhibition or combinational stimulation of lipopolysaccharide (LPS) and interferon-γ induce cleavage of hyper-edited regions located in the 3′ UTRs of CTN-RNA result in the re-polyadenylation and rapid export of the transcripts into the cytoplasm [13]. The transported transcripts then serve as templates for protein synthesis, enabling a rapid response to cellular stress without de novo synthesis of mRNAs. Because SINEs comprise a significant portion of mammalian genomes and are frequently inserted into the 3′ UTR of protein coding genes [51], a large number of genes are expected to be regulated in this manner. In fact, multiple transcripts containing inverted insertions of Alu repeats receive A-to-I editing, and some of their 3′ UTR sequences were experimentally validated to function as nuclear retention signals [52].

Although nuclear-retained CTN-RNAs are proposed to participate in cellular stress responses as mentioned above, the question remains whether any paraspeckle-localizing mRNAs are regulated under normal physiological conditions. Notably, Carmichael and colleagues found that embryonic stem (ES) cells do not contain paraspeckles due to the lack of NEAT1 expression, and nuclear bodies are only formed after the in vitro differentiation of ES cells, which induces the expression of NEAT1 [12]. Consistently, Lin-28 mRNA, which contains inverted SINE repeats in the 3′ UTR that normally function as potent nuclear retention signals in HEK293 cells, is efficiently transported into the cytoplasm in ES cells [12]. These authors also demonstrated that paraspeckles are functionally involved in the nuclear retention of multiple genes with inverted SINE repeats, such as CTN-RNA. When NEAT1 is depleted with antisense oligonucleotides, the amount of target transcript in the nuclear fraction is significantly decreased, and there is a concomitant increase in the cytoplasmic fraction [12]. Considering that paraspeckles are formed upon ES cell differentiation [12], these results imply that paraspeckle-mediated nuclear retention is involved in the maintenance of the undifferentiated state of ES cells and their subsequent differentiation during development.

## Paraspeckle formation in living animals and its physiological function

**Fig. 3:**
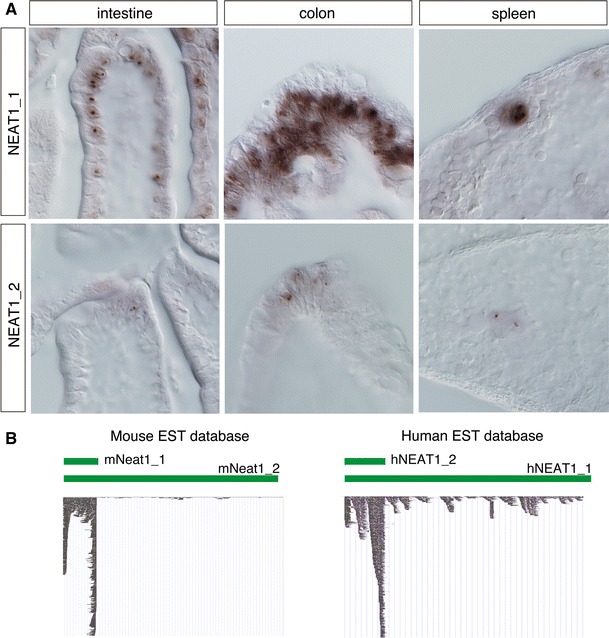
Expression of NEAT1_1 and NEAT1_2 in vivo*.* Both NEAT1 isoforms are ubiquitously expressed in cultured cell lines, but NEAT1_1 is expressed in particular cell types, and NEAT1_2 expression is further restricted to a small number of cells in particular regions. **a** Expression of NEAT1_1/2 in the intestine, colon, and spleen. In the gut, NEAT1_2 is expressed in a region where natural cell loss occurs. In the spleen, only megakaryocytes express NEAT1_2. **b** Distribution of ESTs in the public genome database, UCSC genome browser (http://genome.ucsc.edu/). Much smaller numbers of ESTs are mapped to NEAT1_2 -specific regions

All aforementioned studies were performed using cultured cell lines or primary cultures, and limited northern blot or RT-PCR expression data are available for the tissue distribution of the NEAT1 transcripts. In 2011, S. Nakagawa and colleagues [43] at the RIKEN Advanced Science Institute, Japan, reported that NEAT1_1 is not ubiquitously expressed and that NEAT1_2 expression is further restricted to a limited population of cells in particular tissues (Fig. 3a). The restricted expression of NEAT1_2 is also evident in public expression sequence tag (EST) databases because fewer ESTs are mapped to NEAT1_2-specific 3′ regions (Fig 3b). Consistent with this finding, the prominent paraspeckle formation revealed by the intense accumulation of paraspeckle protein components is not observed in most cells in living animals, although a restricted localization of these marker proteins is frequently found at the NEAT1 transcription site [43]. Considering that NEAT1_1 has the capacity to bind to p54^nrb^ [49], the paraspeckle components are transiently tethered at the transcription site; however, the concentration of these molecules is below the threshold required to generate large, structured paraspeckles that usually bud off from the transcription site and drift into the nucleoplasm.

The restricted formation of paraspeckles in a limited population of cells is rather unexpected because essentially all the cultured cell lines examined this far have expressed NEAT1_1/2, with the exception of ES cells. Interestingly, mouse embryonic cells, which normally do not express NEAT1_2, readily upregulate the expression of this noncoding RNA and form paraspeckles when they are dissociated into single cells and placed in a culture dish [43]. The in vitro culture condition may trigger a certain signaling pathway that leads to the induction of NEAT1_2 expression, which may account for its nearly ubiquitous expression in a variety of cultured cell lines [11]. It should be noted that NEAT1_1 was originally identified by Rangarajan’s laboratory as a gene that is up-regulated upon infection of neural cells with Japanese encephalitis virus or rabies virus in the nervous system, suggesting that paraspeckle formation is conditionally induced when cells are placed under pathogenic conditions [37]. In digestive tissues such as the stomach and colon, strong NEAT1_2 expression and prominent paraspeckle formation is observed in regions where natural cell loss occurs, especially in presumptive pre-apoptotic cells located at the surface-most region of the epithelium facing the lumen [43]. The expression of NEAT1_2 in MEFs is low during early passages and reaches a maximum when these cells become senescent (S.N. unpublished observation). In the spleen, NEAT1_2 expression is found in megakaryocytes that produce platelets (Fig. 3a). In the testes and ovaries, NEAT1_2 is expressed in hormone-producing cells such as Leydig cells and corpus luteal cells [43]. No physiological features are shared between these NEAT1_2-expressing cells, except that they are post-mitotic, terminally differentiated cells. Paradoxically, paraspeckles are not found in dividing cells in living animals, whereas cultured cell lines proliferate and form prominent paraspeckles in vitro. The identification of the precise molecular pathway that leads to the specific production of NEAT1_2 may solve this paradox.

Given that the formation of paraspeckles is conditional, what is the physiological significance of these nuclear bodies? Nakagawa and colleagues [43] generated knockout mice that lack the expression of NEAT1_2 and thus lack paraspeckles. Surprisingly, the knockout mice are viable and fertile, and conventional histological analyses fail to detect any obvious abnormalities [43]. Microarray analyses using cells or tissues derived from the knockout mice also fail to reveal clear changes in gene expression (S.N., unpublished observation), suggesting that paraspeckles are dispensable for regular gene expression under normal laboratory conditions. Considering that NEAT1 expression is induced upon virus infection [37], it would be intriguing to study whether the NEAT1 knockout mice show increased or decreased susceptibility to viral infections. The lack of an apparent phenotype in mice lacking the paraspeckles reminds us of the surprisingly modest phenotype of mouse lacking PML bodies, another type of nuclear body [53]. The PML knockout mice, however, show a defect in DNA-damage-induced apoptosis [53]. Therefore, determining the appropriate assay conditions to fully understand the physiological role of paraspeckles is critical. Considering that paraspeckles are specific to higher eukaryotes, they may not directly regulate essential housekeeping biological processes but rather function as a back-up system to guarantee complex but robust gene regulatory networks.

## Speculation regarding the molecular function of paraspeckles

**Fig. 4:**
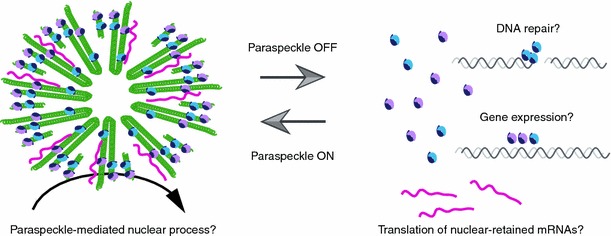
Possible functional mode of paraspeckles. Paraspeckles may function as “positive” nuclear bodies by mediating certain molecular process within the nuclear bodies. Alternatively, paraspeckles may function as “negative” nuclear bodies by sequestering active protein factors that function in other regions of the nucleus

Although we are currently ignorant of the physiological significance of paraspeckle formation in living animals, two types of functional mode can be speculated. First, paraspeckles may serve as “positive” nuclear bodies that regulate certain molecular processes within the nuclear bodies (Fig. 4). As has been proposed, paraspeckles are considered to function as a reservoir for A-to-I edited mRNAs, which are released into the cytoplasm under certain stress conditions. Considering that a wide spectrum of proteins localizes to paraspeckles, certain molecular reactions such as RNA processing/degradation and/or protein modification/degradation may be executed within the nuclear bodies. Interestingly, NEAT1 is processed into small RNAs [54], which might provide another layer of gene expression regulation mediated by the nuclear bodies. The other possibility is that paraspeckles function as “negative” nuclear bodies that indirectly regulate certain molecular processes by controlling the amount of paraspeckle-localizing protein components via sequestration (Fig. 4). We note that all of the currently identified paraspeckle components are diffusely distributed throughout the nucleoplasm with the exception of the architectural lncRNAs, NEAT1_1 and NEAT1_2, which exclusively localize to the paraspeckles. In mouse tissues, the nucleoplasmic level of PSF appears to depend on the level of NEAT1 expression. In cells that abundantly express NEAT1_2, most of the immunofluorescent signals of PSF are observed in paraspeckles, although there is a low background signal in the nucleoplasm. In contrast, the nucleoplasmic signals of PSF are significantly higher in cells that express low levels of NEAT1_2, which lack prominent paraspeckles (S.N., unpublished observation). RNA-dependent sequestering of nuclear proteins is a major cause of neurological diseases, including myotonic dystrophy, in which expanded CUG repeats sequester functional MBNL and CUGBP1 within the nucleoplasm [55]. Similarly, paraspeckle formation might counter-regulate the function of paraspeckle-localizing factors, which normally regulate distinct nuclear processes outside the paraspeckles. In this regard, it is interesting to note that the PSF/p54^nrb^ complex has been identified in a variety of functional biochemical and cell biological assays, including splicing regulation, mRNA stability control, the DNA damage response, transcriptional regulation, and DNA pairing [23–35]. To reveal the physiological function of paraspeckles, addressing the physiological function of the paraspeckle proteins beforehand is essential, although no gene-targeted mice have been reported for any of the core paraspeckle components.

## Closing remarks

Since their discovery in 2002, the knowledge on paraspeckles is accumulating at a rapid rate, especially with respect to their molecular constituents and assembly processes [11]; however, the functional studies are not as advanced. We currently know that animals lacking paraspeckles are normal when raised under laboratory conditions and that paraspeckles are dispensable for most developmental processes. Paraspeckles thus appear to have no function, giving rise to the question as to whether there are any advantages for the animals to retain these apparently useless nuclear bodies during the course of evolution. NEAT1 is conserved in mammalian species but is not easily identified in other vertebrate species [54]. Paraspeckles might thus have played critical roles at the emergence of the common mammalian ancestors, with a function that is no longer essential. Alternatively, we may just be ignorant of the critical conditions required for paraspeckle formation to become critical for cellular function. The real breakthrough will come when we find precise experimental or environmental conditions that allow us to understand the usefulness of what is considered useless.
